# Coagulation factor II thrombin receptor as a promising biomarker in breast cancer management

**DOI:** 10.1515/biol-2022-1001

**Published:** 2024-12-06

**Authors:** Yan-Ming Dong, Guo-Qiang Bao

**Affiliations:** Department of General Surgery, The Second Affiliated Hospital of Air Force Medical University, No. 356 of Xinsi Road, Baqiao District, Xi’an, 710038, China

**Keywords:** breast cancer, dasatinib, F2R, immune cell infiltration, prognosis

## Abstract

This study aims to comprehensively investigate the role of coagulation factor II thrombin receptor (F2R) in breast cancer (BC) and to evaluate its potential as a biomarker in this context. Data on female BC were retrieved from the TCGA database. Comparative analyses were performed, including enrichment analysis, tumor immune microenvironment analysis, drug sensitivity testing, molecular docking, and cell-based experiments, to assess the expression and function of F2R in BC. Statistical analyses and graphical representations were conducted using R software. The study confirmed a significant upregulation of F2R in BC, which was associated with a more favorable prognosis. Clinical correlation analysis revealed a strong association between F2R expression and key clinical parameters, such as estrogen receptor and progesterone receptor status. Additionally, genes co-expressed with F2R were significantly linked to various biological processes, including cell cycle regulation, oxidative phosphorylation, ribosomal function, and extracellular matrix interactions. F2R also showed associations with immune modulators, particularly CD200 and NRP1. Drug sensitivity analysis, molecular docking, and cell experiments consistently demonstrated positive correlations between F2R expression and sensitivity to dasatinib. This study underscores the potential of F2R as a valuable biomarker in BC, providing insights into the molecular mechanisms underlying tumorigenesis.

## Graphical abstract

**Figure:**
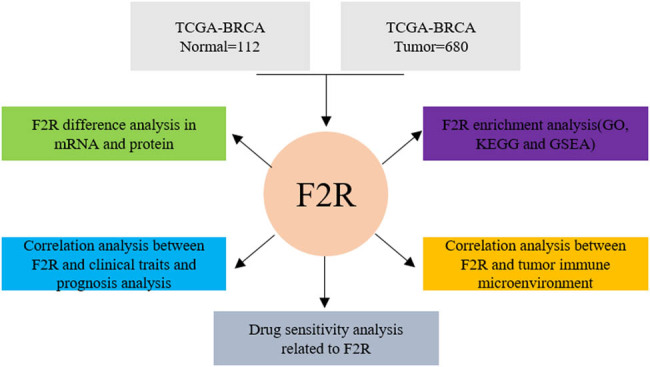

## 1 Introduction

Breast cancer (BC) remains a prevalent malignancy, contributing significantly to global mortality and morbidity rates [1]. China, in particular, has the highest incidence and mortality rates of BC worldwide, accounting for 17.6% of global BC cases and 15.6% of BC-related deaths [2]. Despite advances in detection and treatment, there is a persistent shortage of reliable biomarkers for early screening and diagnosis of BC. The advent of high-throughput sequencing has markedly improved the identification of a wide array of genes that may serve as early-stage markers for BC. For instance, similar to TRIM28, elevated levels of TRIM28 are closely associated with BC metastasis and treatment outcomes [3,4]. The molecular mechanisms underlying these associations are complex and multifaceted [5,6]. Thus, there remains a critical need to identify key driver genes that significantly influence the immune microenvironment and progression of BC. Increasing efforts are being devoted to identifying core genes associated with BC through RNA-seq data from the TCGA database. Detailed analyses of these core genes, exploration of the potential impact of competing endogenous RNA (ceRNA) networks on BC prognosis, and evaluation of immune-targeted drug sensitivity through the TCGA database offer substantial potential for enhancing targeted therapies, prognostic assessments, and diagnostic processes in BC.

Coagulation factor II thrombin receptor (F2R), a member of the protease-activated receptor (PAR) family, functions as an evolutionarily conserved regulator of cell polarity, primarily observed in polarized cells. PAR proteins, including F2R, engage in cross-regulatory interactions with other polarity complexes, contributing to the establishment of stable cellular asymmetry. These interactions also influence the distribution of PAR proteins in response to stimuli such as cytoskeletal transport, reflecting their dynamic nature throughout evolution [7]. Previous studies have demonstrated increased F2R expression in various tumors, including lung adenocarcinoma, glioma, melanoma, and gastric cancer [8,9,10,11,12]. In these contexts, elevated F2R expression has been closely linked to the biological progression of malignancies, including angiogenesis, invasion, and proliferation. However, research on the role of F2R in BC remains limited. The present study aims to investigate the prognostic implications and underlying mechanisms of F2R in BC.

Using bioinformatics analyses of sequencing data from the TCGA database, we examined regulatory pathways and disease networks associated with BC. A comparative analysis of differentially expressed genes (DEGs) in individuals with BC was performed using specimen data from the TCGA. The expression profiles were correlated with survival outcomes. RNA analysis included specimens from 680 individuals with BC and 112 healthy controls. To further elucidate the biological role of F2R in BC, gene set enrichment analysis (GSEA) and Gene Ontology (GO)/Kyoto Encyclopedia of Genes and Genomes (KEGG) enrichment analysis were employed to uncover underlying molecular interactions. A systematic screening of microRNAs (miRNAs) and long noncoding RNAs (lncRNAs) upstream of F2R was conducted, establishing a ceRNA network to clarify F2R’s potential regulatory role in BC development. Additionally, the prognostic significance of F2R in BC was investigated, along with a comprehensive evaluation of its potential clinical applications. The findings of this study contribute to the identification of new prognostic biomarkers and provide insights into mechanisms that may influence BC prognosis. *In vitro* experiments were also conducted to support these findings.

## 2 Materials and methods

### 2.1 Data collection and screening

RNA-seq data and clinical information for BC were retrieved from the TCGA database. After excluding samples with incomplete clinical information, the final dataset comprised 680 samples from individuals with BC and 112 samples from healthy individuals as controls. Detailed information on the 680 individuals with BC is provided in Table 1.

**Table 1:** Clinical characteristics of 680 patients with BC

Characteristics	*N* (%)
**Age**	
≤65	494 (72.65)
>65	186 (27.35)
**Stage**	
I	116 (17.06)
II	396 (58.24)
III	160 (23.53)
IV	8 (1.18)
**T classification**	
T1	172 (25.29)
T2	403 (59.26)
T3	87 (12.79)
T4	18 (2.65)
**N classification**	
N0	331 (48.68)
N1	221 (32.50)
N2	84 (12.35)
N3	44 (6.47)
**PR**	
Negative	226 (33.24)
Positive	454 (66.76)
**ER**	
Negative	156 (22.94)
Positive	524 (77.06)
**Her-2**	
Negative	528 (77.65)
Positive	152 (22.35)
**Survival status**	
Death	70 (10.29)
Alive	610 (89.71)

### 2.2 Differential expression analysis of F2R in BC

The TIMER database, which includes comprehensive genetic data from various sources such as the TCGA, encompassing a total of 10,897 samples [13], was utilized to perform a pan-cancer analysis of F2R expression. Expression data for F2R were extracted and analyzed across the 680 samples from patients with BC, 112 healthy controls, and 112 paired samples using the “Limma” R package [14].

### 2.3 F2R protein expression level validation

To validate F2R protein expression levels in both BC and healthy control tissue specimens, immunohistochemistry (IHC) data from the Human Protein Atlas (HPA) website (https://www.proteinatlas.org/) were analyzed.

### 2.4 Correlation analysis between F2R and clinical traits

Clinical data from patients with BC, including parameters such as T/N stage, overall stage, PR/ER/Her-2 status, and age, were integrated with F2R expression levels for a comprehensive analysis. Logistic regression analysis was conducted using R (Version 4.3.2) to assess the relationship between clinical parameters in patients with BC and F2R expression. A *p*-value of less than 0.05 was considered statistically significant.

### 2.5 Prognostic analysis

The “Survival” and “Survminer” R packages were employed to investigate the correlation between BC survival outcomes and F2R expression. Further validation was conducted using the KM plotter database [15]. The results are presented using survival curves, including log-rank *p*-values and hazard ratios (HR).

### 2.6 Functional enrichment analysis

GO, KEGG, and GSEA analyses were conducted to assess the functional pathway enrichment associated with F2R. These analyses were conducted using the R packages “Clusterprofiler” and “org.Hs.eg.db” [16]. A significance threshold of *p*-value <0.05 was applied for the analyses.

### 2.7 Immune cells infiltration analysis

To quantitatively assess the relative expression levels of F2R across 28 immune cell types in humans, single-sample genome enrichment analysis was conducted using the R package “GSVA” [17]. Additionally, the infiltration levels of 22 distinct immune cell types in lung cancer were quantified using the R package “CIBERSORT.” A significance threshold of *p* < 0.05 was applied to both analyses.

### 2.8 ceRNA regulatory network construction

The interactions between F2R mRNA and miRNAs were analyzed using the miRDB, miRanda, and TargetScanHuman databases. Only mRNA–miRNA pairings supported by all three databases were retained for further analysis. Subsequently, the spongeScan online database was employed to predict lncRNA-miRNA pairs based on the previously identified F2R mRNA-miRNA interactions. The interactive relationships among F2R mRNA, miRNAs, and lncRNAs were visualized using Cytoscape 3.10.2 software.

### 2.9 Therapeutic analysis

BC specimens were categorized into low and high F2R expression groups based on the median value of F2R expression. The Genomics of Drug Sensitivity in Cancer (GDSC) database and the “pRRophetic” package were utilized to assess tumor chemosensitivity [18]. The IC_50_ values of chemotherapeutic drugs were determined using a regression-based approach, with the accuracy of the regression model assessed through 10-fold cross-validation on the GDSC training dataset. Default parameters were applied, including “combat” for batch effect correction and the averaging of replicate gene expression data. Additionally, gene expression data from the Cancer Cell Line Encyclopedia (CCLE) and drug sensitivity data from the CellMiner database were used to investigate the relationship between F2R expression and drug responses in tumor cell lines [19].

### 2.10 Molecular docking

Molecular docking was performed to explore the interaction between F2R and candidate drugs. The molecular structures of the drugs were obtained from PubChem, while the 3D protein structure of F2R was sourced from the Protein Data Bank (PDB). Using PyMOL software, the initial ligands and water molecules were removed from the target protein structure. The target proteins were then prepared for docking by hydrogenation, charge calculation, and nonpolar hydrogen combination using AutoDock Tools 1.5.6. Docking analysis between F2R and the drugs was conducted using AutoDock Vina 1.2.0 software [20]. The docking results were saved in PDBQT format and visualized using PyMOL software.

### 2.11 Cell culture

In this study, both healthy breast epithelial cells (MCF-12A) and BC cells (MCF-7) were cultured in DMEM supplemented with 10% fetal bovine serum at 37°C with 5% CO_2_. Experimental procedures were carried out using cells in the logarithmic growth phase.

### 2.12 Quantitative PCR

Total RNA was extracted using TRIzol reagent (Invitrogen, USA) according to the manufacturer’s instructions. cDNA synthesis was subsequently performed using a corresponding kit (Ambion; Thermo Fisher Scientific, Inc.), following the recommended protocol. Quantitative PCR (qPCR) was conducted using a commercially available kit, with each experiment replicated three times for consistency. The primers used for qPCR were as follows F2R: forward sequence: 5ʹ-CCACCTTAGATCCCCGGTCAT-3ʹ, reverse sequence: 5ʹ-GTGGGAGGCTGACTACAAACA-3ʹ. GAPDH: forward sequence: 5ʹ-AAAGCCTGCCGGTGACTAA-3ʹ, reverse sequence: 5ʹ-AGAGTTAAAAGCAGCCCTGG-3ʹ. GAPDH was utilized as the reference gene for normalizing the mRNA expression levels of F2R.

### 2.13 Cell proliferation assay

Cell viability was assessed using the MTT assay, following standard protocols. Optical density at 490 nm (OD490) was measured at specified time points. Cells were treated with various concentrations of dasatinib (Selleck, S1021), ranging from 50 μM/L to 0 μM/L. The ratio of cell viability (%) was calculated using the formula: (Test group OD490 − Empty control OD490)/(Control group OD490 − Empty control OD490) × 100%. The half-maximal inhibitory concentration (IC_50_) was determined using GraphPad Prism 8.0 software.

### 2.14 Cell migration assays

Cell migration ability was assessed using a wound-healing assay. Cells were seeded in 6-well plates and allowed to reach approximately 100% confluence. A sterile (yellow) pipette tip was used to create a straight scratch across the cell monolayer. Following this, 6 μM/L dasatinib was added to the plates. The initial scratch distance (D0) and the distance at 48 h (D48) were measured using a Nikon TS100 microscope. The cell migration distance at 48 h was calculated as the difference between D0 and D48. The 48-h cell migration ratio (%) was calculated as the 48-h cell migration distance divided by D0, multiplied by 100%.

### 2.15 Measurement of F2R

F2R levels were measured using the Human PAR1 ELISA Kit (Invitrogen, EH358RB) following the standard protocols. A 100 μL protein sample was added to each well of a micro-ELISA plate and incubated for 2.5 h at room temperature. Subsequently, 100 μL of biotinylated detection F2R antibody was added to each well and incubated for 1 h at room temperature with gentle shaking. Following this, 100 μL of HRP-conjugated diluent was added to each well and incubated for 45 min at room temperature with gentle shaking. Next, 100 μL of tumor mutational burden (TMB) substrate was added, and the plate was incubated for 30 min at room temperature in the dark with gentle shaking. Finally, the reaction was terminated by adding 50 μL of stop solution, and the absorbance was promptly measured at *λ* = 450 nm within a strict 2-min timeframe.

### 2.16 Statistical analysis

Statistical analysis was performed using GraphPad Prism 9.0 and R (version 4.3.3), utilizing the ggpubr, ggplot2, and limma packages for data analysis and visualization. The Wilcoxon signed-rank test was used to evaluate F2R expression in paired samples, while the Wilcoxon rank-sum test was employed for unpaired samples. The relationship between clinical features and F2R expression was analyzed using the limma package, with ggpubr employed for generating graphs. Survival outcomes were evaluated using Kaplan–Meier (K–M) analysis. Additionally, CIBERSORT was utilized to estimate the abundances of 22 infiltrating immune cell types within the tumor microenvironment (TME) based on gene profiles of patients with BC obtained from TCGA. Concurrently, the ESTIMATE algorithm was utilized to derive matrix and immune scores from the same datasets, which were then graphically represented. Stromal and immune scores were compared across various clinicopathological cohorts using the Wilcoxon test, with statistical significance defined as *p* < 0.05.

## 3 Result

### 3.1 Disparities in F2R expression patterns between BC and other cancer types

**Figure 1:**
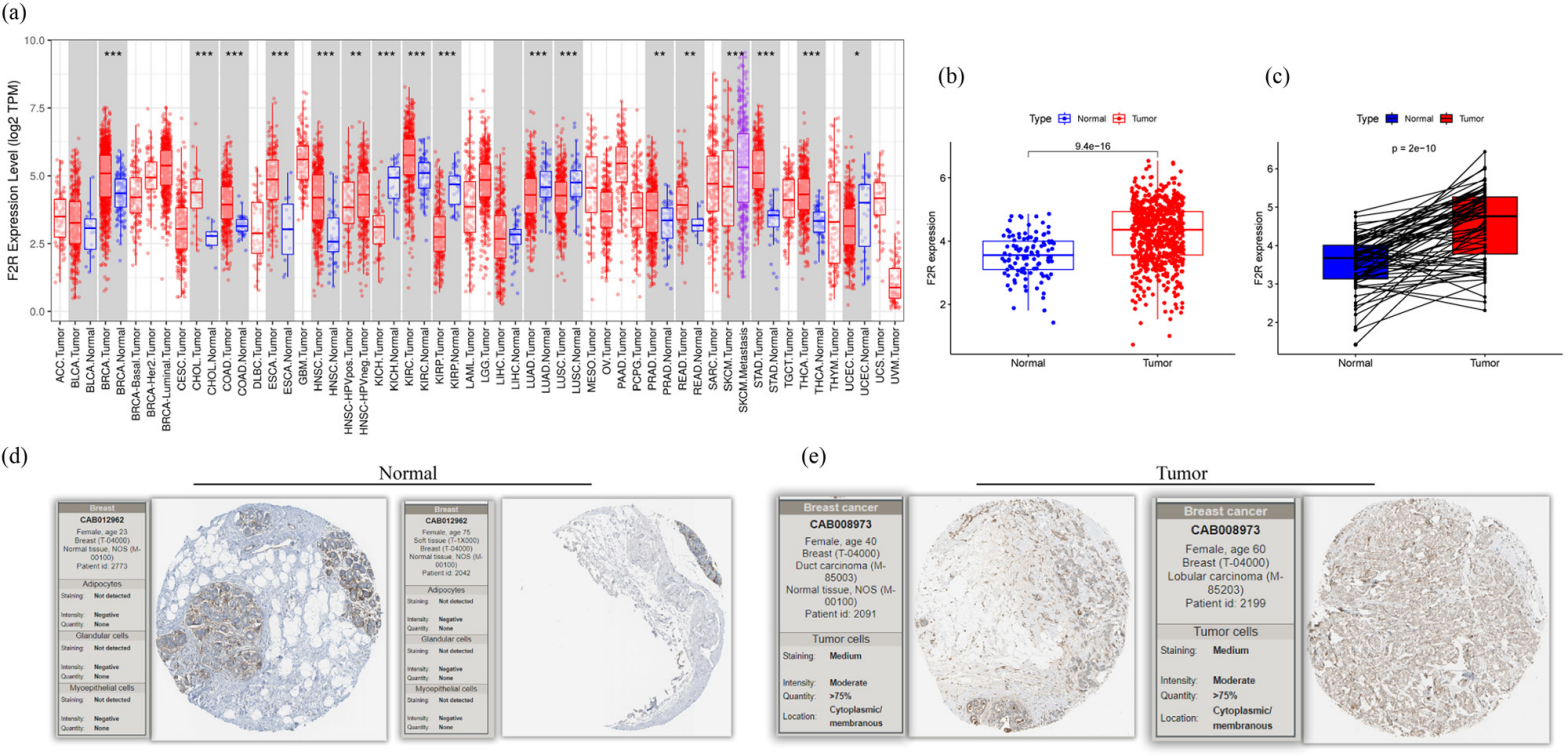
Significant increase in F2R expression level in BC samples. (a) F2R expression across various tumor tissues in the TCGA database analyzed using the online TIMER tool. (b) and (c) F2R expression elevated in BC based on the TCGA data set. (d) and (e) F2R protein expression in both normal and tumor tissues of BC examined using immunohistochemistry (IHC) data.

To explore the differences in F2R expression between tumor and healthy tissues, F2R mRNA levels were analyzed across various cancer types and their corresponding controls using data from the TIMER database. In addition to BC, significant elevations in F2R mRNA levels were observed in several other cancer types, including colon cancer, stomach cancer, head and neck cancer, lung adenocarcinoma (LUAD), thyroid cancer, bile duct cancer, esophageal cancer, lung squamous cell carcinoma (LUSC), kidney clear cell carcinoma (KIRC), and kidney chromophobe (KICH). However, there were significantly lower levels in LUAD, KICH, and LUSC compared to their respective healthy tissues (Figure 1a).

Further analysis of F2R mRNA expression was conducted in 680 patients with BC and 112 healthy controls with complete clinical data. The results revealed a notable increase in F2R expression levels in BC samples compared to the control group (Figure 1b). In paired samples, the analysis consistently demonstrated higher levels of F2R in the tumor group compared to the corresponding control group (Figure 1c). Additionally, F2R protein expression was examined in both healthy and BC tumor tissues using IHC data. The analysis revealed distinct F2R staining patterns, with weak F2R staining observed in healthy tissues and notably strong F2R staining evident in tumor tissues (Figure 1d–e).

### 3.2 F2R expression and its relationship to patient prognosis and clinicopathological characteristics in BC

**Figure 2:**
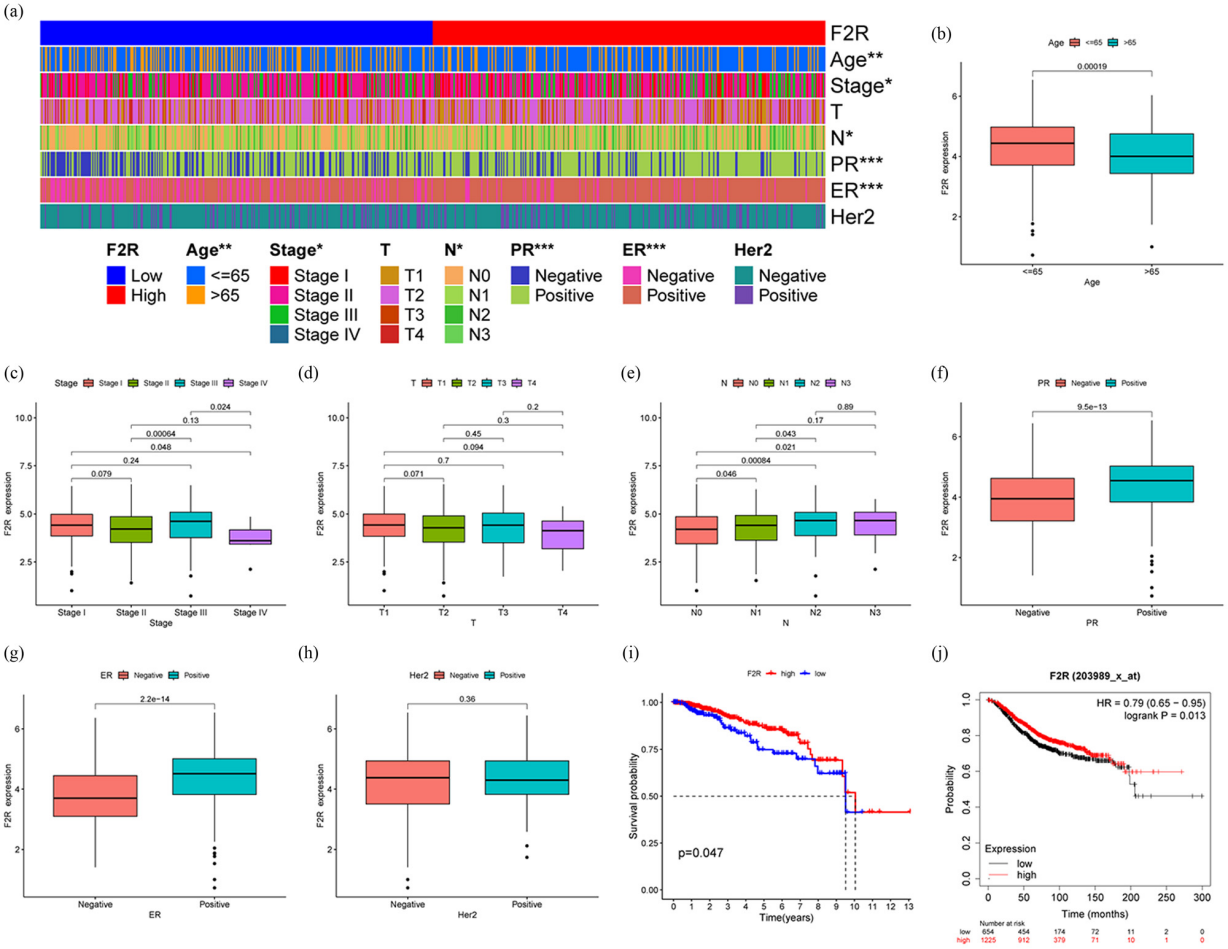
Expression of F2R correlates with the prognosis and clinicopathological characteristics of patients with BC. (a) Clinical data analysis using thermal imaging in patients with BC with varying levels of F2R expression reveals a significant correlation between F2R expression and key clinicopathological parameters such as age, stage, N stage, PR status, ER status, and Her2 status. (b)–(h) Furthermore, F2R expression is positively correlated with histological grade, pathological stage, T stage, and N stage, but not with age, gender, or M stage. (i) Analysis of TCGA dataset demonstrates that patients with BC with high F2R expression exhibit significantly longer overall survival compared to those with low F2R expression. (j) The prognostic significance of F2R in BC is confirmed through validation using data from the K–M Plotter website. *p < 0.5, **p < 0.01, ***p < 0.001.

The relationship between F2R gene expression and clinicopathological variables in patients with BC was further investigated. Patients were divided into two groups based on their F2R expression levels, and relevant parameters were analyzed alongside dichotomized F2R expression (high/low) using thermographic analysis. Statistically significant associations were found between F2R expression and patient age, clinical stage, N stage, progesterone receptor (PR) status, and estrogen receptor (ER) status (*p* < 0.05) (Figure 2a).

Subsequently, F2R mRNA expression was assessed across groups categorized by age, clinical stage, T/N stages, and ER/PR/Her-2 status. The results demonstrated a significant association between F2R overexpression and age (≤65 and >65, *p* = 0.00019), PR status (negative and positive, *p* = 9.5 × 10^−13^), as well as estrogen receptor (ER) status (negative and positive, *p* = 2.2 × 10^−14^) (Figure 2b–h). Notably, F2R gene expression was found to be influenced by clinical factors such as age, PR, and ER. K–M survival curves indicated that patients with high F2R expression had longer survival compared to the low F2R group (*p* = 0.047) (Figure 2i). These findings were corroborated using the K–M Plotter, which confirmed that patients with BC with high F2R expression experienced longer survival times (Figure 2j).

### 3.3 F2R‑related pathways evaluated using GSEA, GO, and KEGG

**Figure 3:**
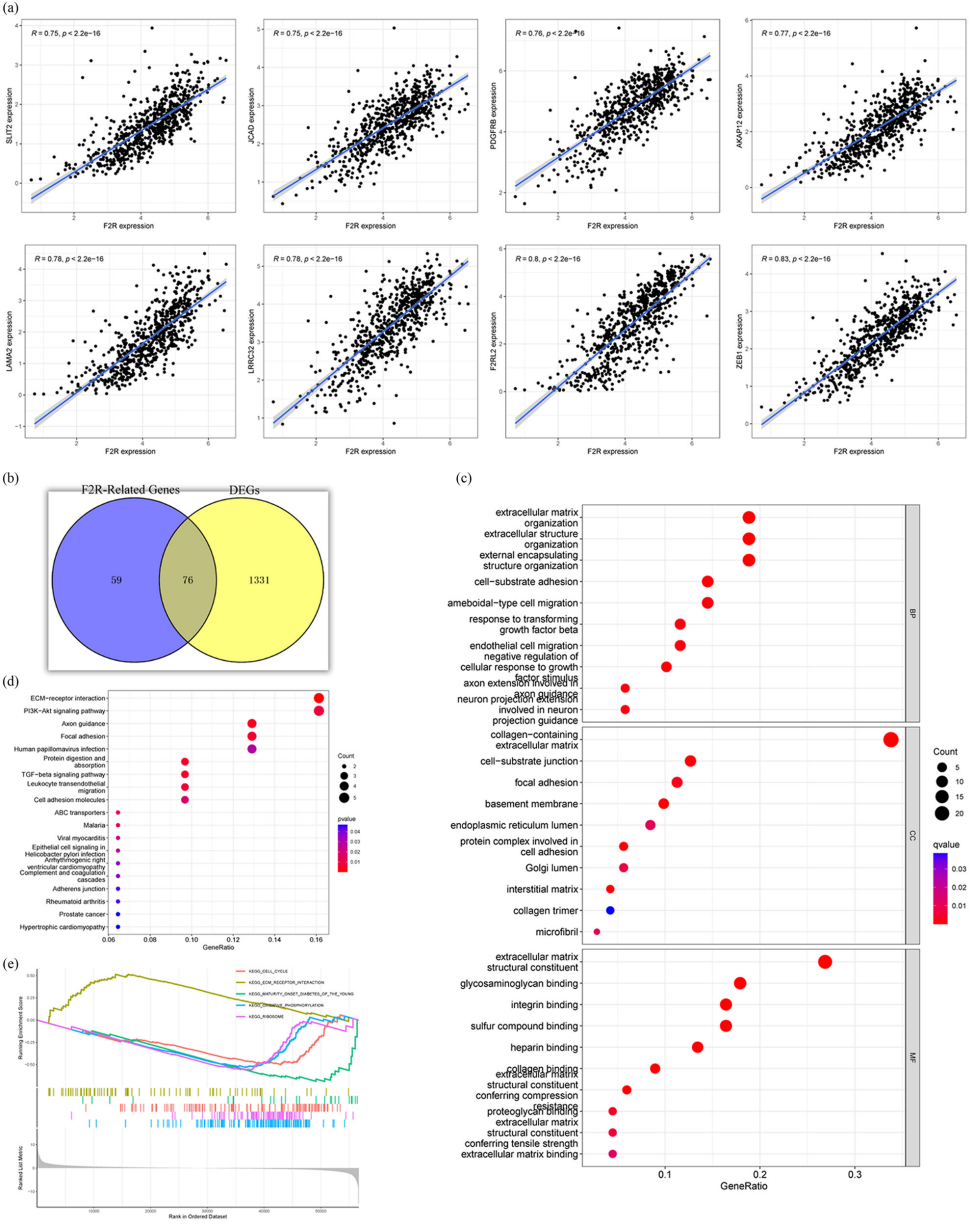
Gene enrichment analysis to identify pathways associated with F2R. (a) Genes highly correlated with F2R (correlation coefficient >0.6) were identified in BC using Pearson’s correlation analysis. (b) A subset of 76 DEGs exhibiting a strong correlation with F2R were selected for further investigation. (c) Gene ontology (GO) enrichment analysis was performed to explore the functional characteristics of F2R-related DEGs. (d) KEGG enrichment analysis was conducted to identify pathways associated with F2R-related DEGs. (e) GSEA was utilized to elucidate the signal pathways associated with F2R mRNA expression in BC.

To comprehensively investigate the role of F2R in BC, genes strongly associated with F2R (correlation coefficient >0.6) were screened in BC using the Pearson’s correlation analysis. The representative F2R-related genes are depicted in Figure 3a. A total of 1,407 DEGs were identified between the high- and low-expression F2R groups, categorized by the median expression value of F2R under the condition of false discovery rate <0.05 and |LogFC| > = 1.

Subsequently, a subset of 76 DEGs, which displayed a robust correlation with F2R, was selected for in-depth investigation (Figure 3b). This selection focused on genes most closely associated with F2R, allowing for a more detailed examination of their potential roles and interactions in BC. To elucidate the functional significance of selected F2R-related markers, comprehensive analyses were conducted using GO and KEGG pathway enrichment methods.

GO analysis revealed that DEGs associated with F2R were primarily enriched in categories related to cell communication. Notable terms included “external encapsulating structure organization,” “extracellular matrix organization,” “extracellular structure organization,” “response to transforming growth factor beta,” and “cell–substrate adhesion,” indicating a potential role in mediating cell–cell interactions (Figure 3c). KEGG analysis provided additional insights, highlighting enrichment in numerous pathways pivotal for cell regulation and communication. Notable terms included “external encapsulating structure organization,” “extracellular matrix organization,” “extracellular structure organization,” “response to transforming growth factor beta,” and “cell–substrate adhesion,” indicating a potential role in mediating cell-cell interactions (Figure 3c).

To further investigate the potential modulatory mechanisms of F2R in BC, GSEA was conducted. The results indicated that F2R mRNA expression is associated with key biological processes and pathways, including the cell cycle, ECM receptor interaction, ribosome, Parkinson’s disease, DNA replication, and oxidative phosphorylation (Figure 3e).

### 3.4 Correlation between immune cell infiltration and F2R expression

**Figure 4:**
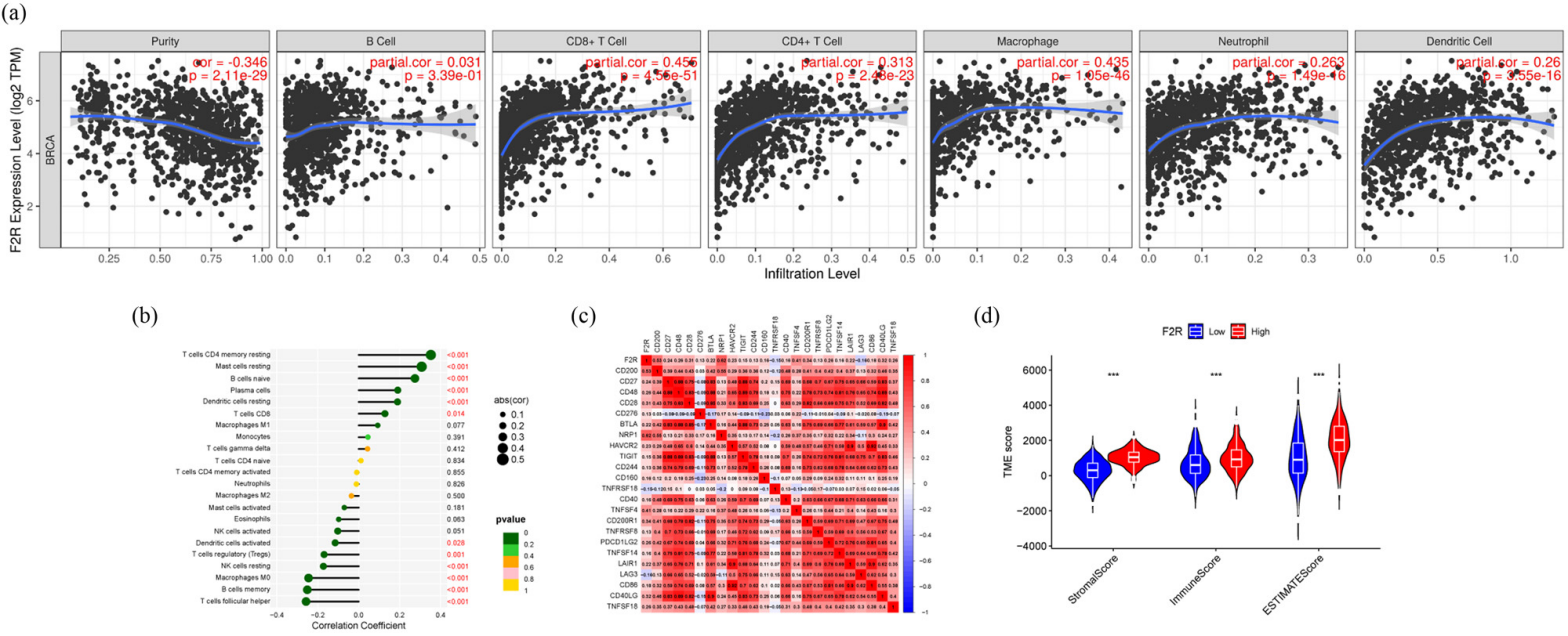
Potential involvement of F2R in immune regulation and infiltration within cancerous tissues. (a) By utilizing the online TIMER tool, correlation analysis was conducted between F2R expression levels and diverse immune cells in BC tissues. (b) CIBERSORT was employed to assess the correlation between F2R expression and 22 immune cell types across 680 samples of BC. (c) Examination of the correlation between F2R expression and immune checkpoint genes was conducted. (d) The expression levels of F2R in BC were categorized into low and high groups, followed by the analysis of immune infiltration using Stromalscores and Immunocore in both groups. ***p < 0.001.

The composition of immune cells in the TME and their correlation with F2R expression in BC were analyzed using data from the TIMER database, as illustrated in Figure 4a. Patients with BC were then categorized into low and high F2R expression groups. The relationship between F2R expression and immune cell infiltration was examined using CIBERSORT, revealing significant differences in immune cell abundances between the two groups. Notably, the high F2R expression group showed a marked increase in the abundance of CD4 memory cells, mast cells, B cells, plasma cells, and dendritic cells. In contrast, the low F2R expression group had higher levels of T follicular helper cells, memory B cells, M0 macrophages, and NK cells (Figure 4b).

A linear relationship analysis between immune checkpoint genes (ICPs) and F2R expression revealed a positive modulatory association, indicating that F2R expression may influence the regulation of ICPs (Figure 4c). Additionally, an analysis of stromal and immune cell infiltration across the groups revealed significant differences in stromal/immune/ESTIMATE scores, all of which were markedly higher in the high F2R expression group (Figure 4d).

**Figure 5:**
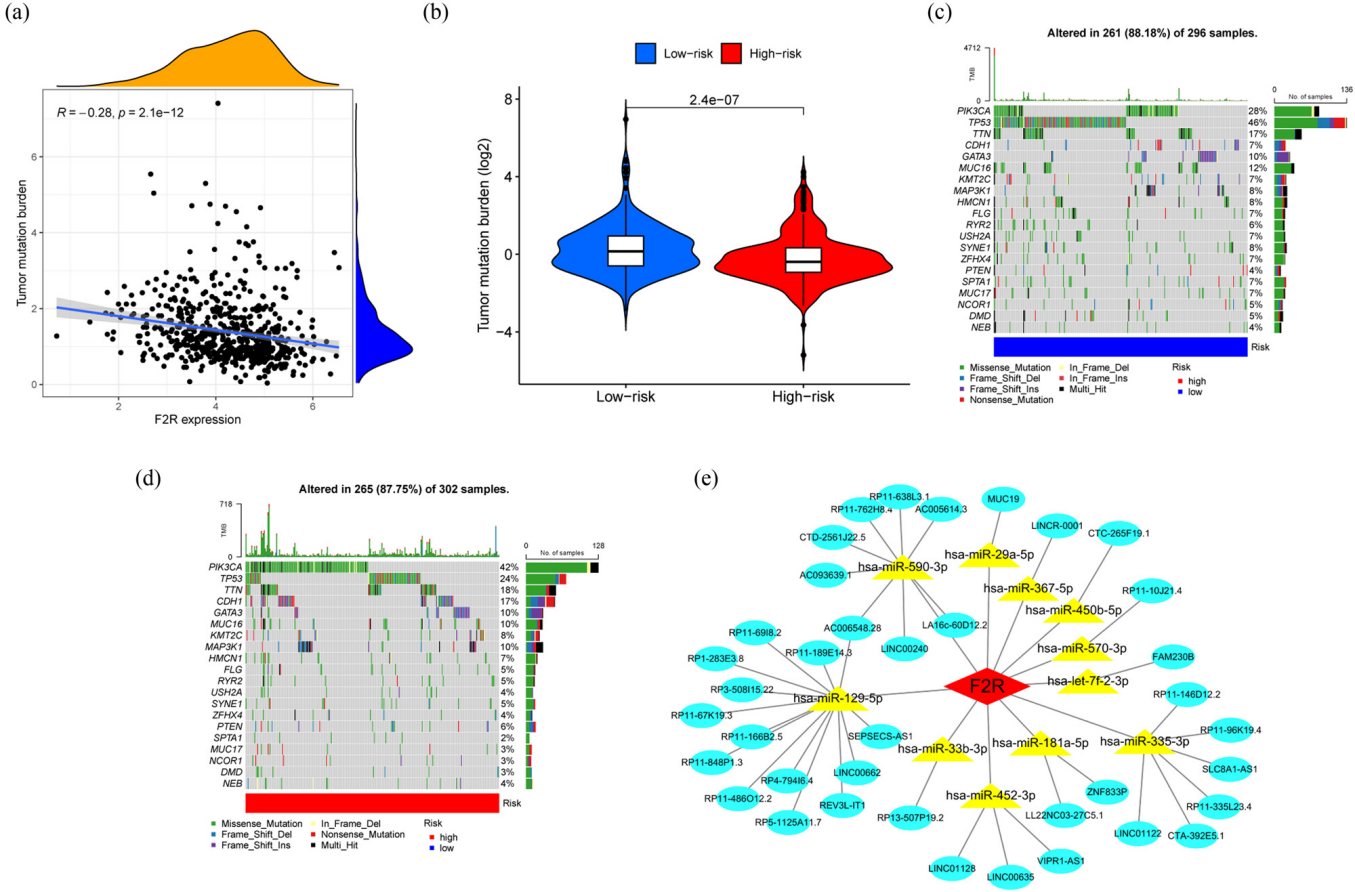
Association between F2R expression and TMB. (a) Elevated F2R expression in BC correlates with decreased TMB. (b) Elevated F2R expression in BC is correlated with decreased TMB. (c) and (d) Waterfall plot illustrating gene mutations in low and high F2R expression groups in BC samples. (e) Identification of upstream miRNAs and lncRNAs regulating F2R expression and construction of the ceRNA network diagram.

Given the critical role of TMB in tumorigenesis and progression, the relationship between F2R expression and TMB was also investigated, along with the relationship between F2R expression and specific gene mutations in BC. The results demonstrated a significant association, with higher F2R expression in BC corresponding to lower TMB (Figure 5a). Furthermore, when comparing the low and high F2R expression groups, the high F2R expression group exhibited reduced TMB (Figure 5b). Analysis of specific gene mutations revealed notable differences in the frequency of mutations in genes such as PIK3CA, TP53, TTN, and CDH1 between the high and low F2R expression groups (Figure 5c and d).

### 3.5 LncRNA–miRNA–mRNA ceRNA regulatory network

Based on the ceRNA hypothesis, which suggests that miRNAs regulate gene expression by targeting mRNA, and lncRNAs modulate this process by binding to miRNAs, a ceRNA network targeting F2R was constructed using bioinformatic approaches. As depicted in Figure 5e, the ceRNA regulatory network includes 11 miRNA nodes. F2R was identified as being regulated by the following miRNAs: hsa-miR-590-3p, hsa-miR-29a-5p, hsa-miR-367-5p, hsa-miR-450b-5p, hsa-miR-570-3p, hsa-let-7f-2-3p, hsa-miR-129-5p, hsa-miR-33b-3p, hsa-miR-181a-5p, hsa-miR-335-3p, and hsa-miR-452-3p. These miRNAs were, in turn, regulated by various lncRNAs.

### 3.6 Correlation analysis between drug sensitivity and F2R expression

**Figure 6:**
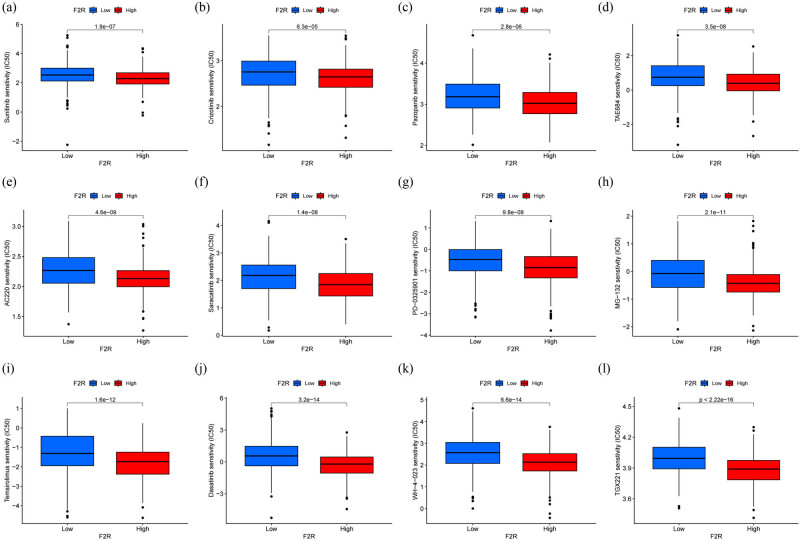
Predicted drug responsiveness in low and high F2R expression groups. (a)–(l) Exemplary drugs displaying heightened sensitivity in high-F2R expression groups, encompassing sunitinib, crizotinib, pazopanib, TAE684, AC220, saracatinib, PD-0325901, MG-132, temsirolimus, dasatinib, WH-4-023, and TGX221.

Numerous studies have focused on identifying biomarkers associated with susceptibility and resistance to anticancer drugs. In this study, sensitivity in BC was analyzed by comparing high and low F2R expression groups. The relationship between F2R expression and IC_50_ values for commonly used BC treatments was evaluated using the pRRophetic R package. The analysis revealed a positive correlation between F2R expression and sensitivity to several chemical compounds, including sunitinib, crizotinib, pazopanib, TAE684, AC220, saracatinib, PD−0325901, MG−132, temsirolimus, dasatinib, WH−4−023, and TGX221 (Figure 6a–l).

**Figure 7:**
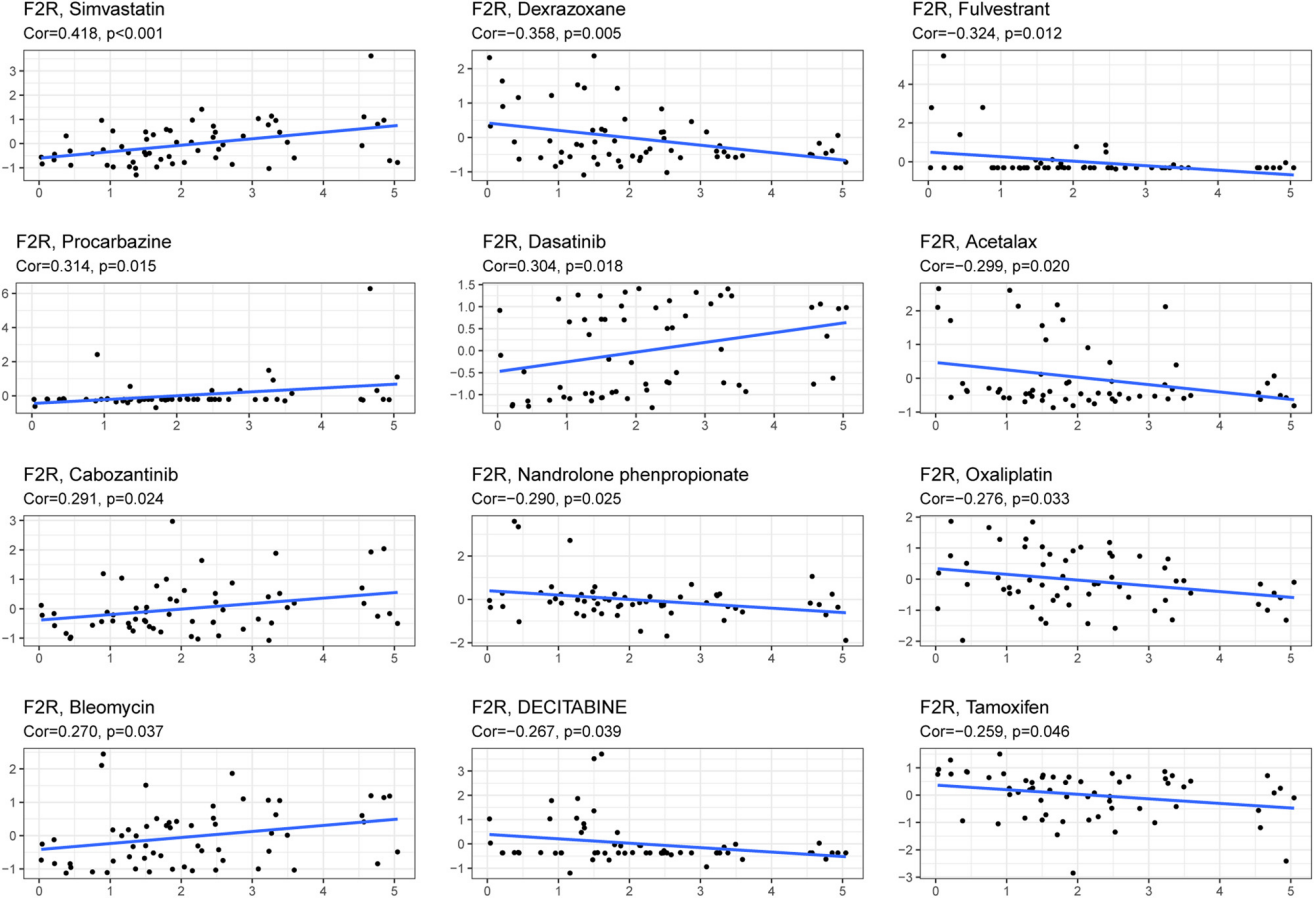
Correlation plot illustrating the relationship between F2R gene expression and drug sensitivity in tumor cell lines. Positive correlation indicates that higher F2R expression is associated with increased drug sensitivity, while negative correlation suggests that higher F2R expression is linked to decreased drug sensitivity. The *x*-axis represents gene expression levels, while the *y*-axis represents drug sensitivity.

Further analysis was conducted using data from the CCLE to explore the relationship between F2R expression and drug sensitivity. The CellMiner database was utilized to assess F2R responsiveness to commonly used antitumor drugs. Correlation analysis indicated a significant association between F2R expression and the sensitivity of 12 drugs (Figure 7). Specifically, F2R expression positively correlated with sensitivity to simvastatin, procarbazine, and dasatinib, while showing a negative association with dexrazoxane and fulvestrant. It is important to note that some of the drugs demonstrating sensitivity to F2R expression have not undergone extensive clinical testing. Further research is required to fully evaluate their potential as viable candidates for therapeutic interventions.

### 3.7 Molecular docking results

**Figure 8:**
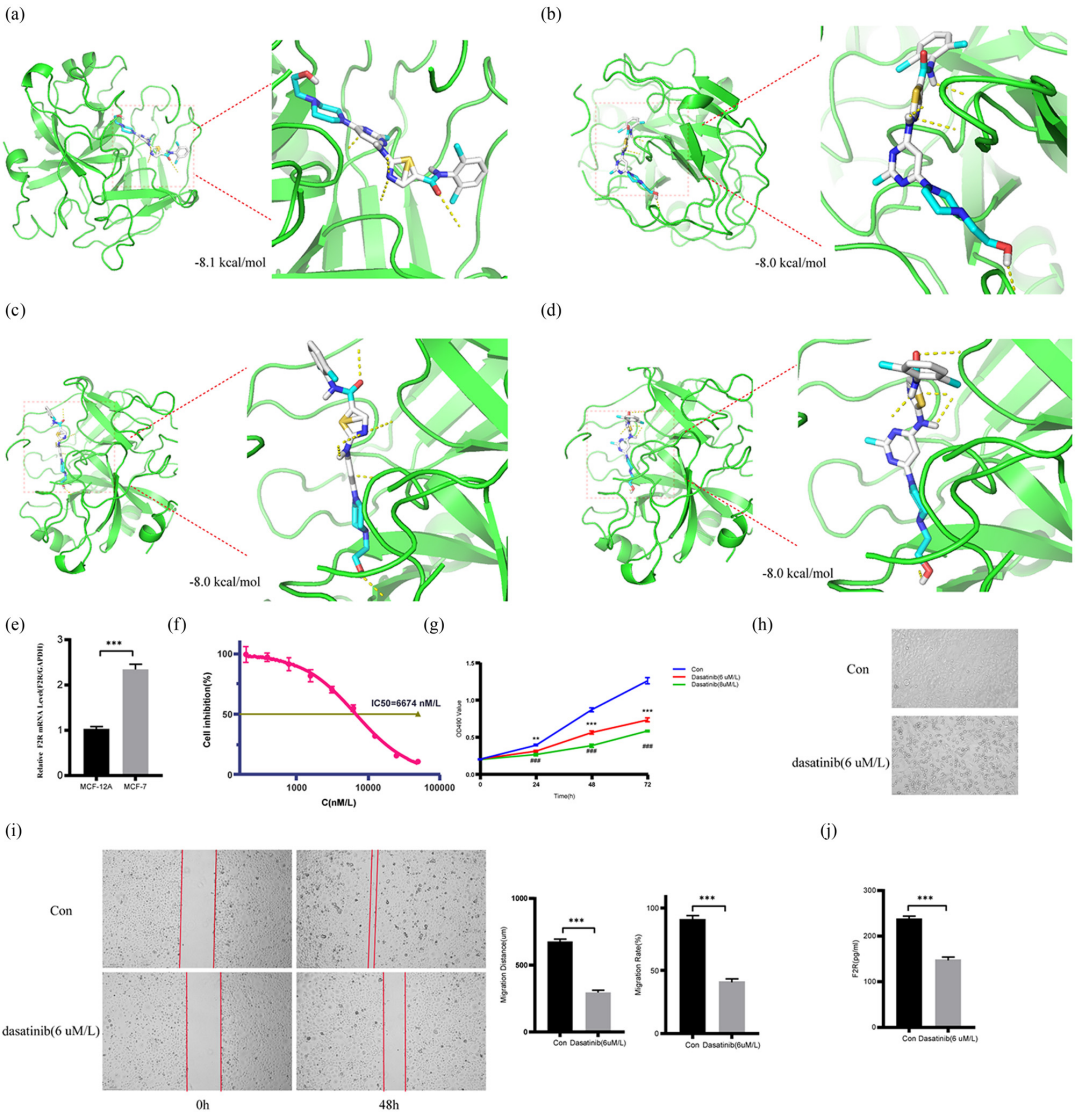
Molecular docking analysis to assess the interaction between F2R and dasatinib and an evaluation of dasatinib’s impact on MCF-7 cells. (a)–(d) Top four molecular docking sites between F2R and dasatinib. (e) Comparison of F2R mRNA expression levels in MCF-12A and MCF-7 cells. (f)–(j) The MTT assay was employed to determine the IC_50_ of dasatinib in MCF-7 cells and assess cell viability following treatment with 6 and 8 μM/L dasatinib. Additionally, the cellular state of MCF-7 cells treated with 6 μM/L dasatinib for 48 h was observed, and a scratch assay was conducted to evaluate the migration ability of MCF-7 cells treated with the same concentration of dasatinib for 48 h. Furthermore, the levels of F2R protein in MCF-7 cells after treatment with 6 μM/L dasatinib for 48 h were measured using the F2R ELISA Kit. **p < 0.01, ***p < 0.001, ###*p* < 0.001.

Given the significant correlation between F2R expression and sensitivity to dasatinib, molecular docking experiments were conducted to analyze the interaction between F2R and dasatinib. The results indicated that stronger binding correlates with lower energy levels between the two entities. The top four molecular docking sites demonstrated successful binding within the docking pocket, suggesting favorable docking activity between the target proteins. Specifically, the binding energy values of dasatinib and F2R were all ≤−8 kcal/mol, with the lowest binding free energy recorded at −8.1 kcal/mol (Figure 8a–d).

### 3.8 High expression of F2R in MCF-7 cells and its association with dasatinib in regulating cell proliferation and migration

To verify F2R expression in BC, experiments were conducted using MCF-7 cells. qRT-PCR demonstrated a significant upregulation of F2R expression in MCF-7 cells compared to MCF-12A (Figure 8e), consistent with the IHC analysis results (Figure 1d–e). To evaluate the cytotoxic impact of dasatinib on BC cell proliferation, MCF-7 cells were treated to varying concentrations of dasatinib for 24, 48, and 72 h, and cell viability was assessed using the MTT assay. The results indicated that dasatinib’s inhibitory effect on cell growth was concentration-dependent. At the highest concentration tested (50,000 nM), dasatinib significantly inhibited cell growth. The IC_50_ of dasatinib in MCF-7 cells was determined to be 6,674 nM/L (Figure 8f). Cells treated with dasatinib showed a significant decrease in cell survival compared to the control group (Figure 8g), underscoring the effectiveness of dasatinib in inhibiting cell growth. Representative images illustrating the cellular states are presented in Figure 8h.

Furthermore, dasatinib effectively inhibited cell migration compared to the control group, as shown in Figure 8i. Notably, intracellular F2R protein expression levels decreased in cells treated with dasatinib for 48 h compared to the control group (Figure 8j). These *in vitro* experiments underscore the potent biological activity of dasatinib against MCF-7 cells and suggest a strong association between F2R expression and the regulation of MCF-7 cell migration, invasion, and proliferation. The findings support the potential of F2R as a novel therapeutic target, particularly in BC, offering promising opportunities for inhibiting tumor progression and advancing targeted therapies in BC treatment.

## 4 Discussion

The exploration of biomarkers and molecular targets is pivotal for the early detection and treatment of BC. Through comprehensive bioinformatics analysis of TCGA data, we identified genes associated with BC pathogenesis and prognosis, notably revealing a significant increase in F2R expression. This finding prompted further investigation into F2R’s role in BC initiation and progression. The elevated expression of F2R in BC, compared to other tumor types, suggests that F2R may have a unique function within the biological context of BC. Understanding whether the role of F2R in BC significantly differs from its functions in other cancer types is essential for unraveling the complexities of BC and could pave the way for personalized therapeutic strategies.

The prognostic value of F2R in BC introduces a novel approach to patient classification and treatment strategies. Our study found a strong correlation between high F2R expression and improved survival outcomes, suggesting that elevated F2R expression is associated with a favorable prognosis in BC. This trend is consistent with observations in liver cancer, where high F2R expression also correlates with a positive prognosis [21]. However, in other cancers such as pancreatic cancer and gastric cancer, high F2R expression is associated with poor prognosis [22,12]. The differences in the prognostic implications of F2R expression across various tumor types can be attributed to variations in the aggressiveness of these malignancies. BC and liver cancer, which are generally less aggressive than gastric and pancreatic cancers, may exhibit different responses to F2R expression.

The high F2R expression in BC could broaden the range of available treatment options, potentially enhancing drug sensitivity and treatment efficacy, and thereby improving patient survival. Elevated F2R expression in BC tissues may act as a barrier to malignant progression, thus prolonging survival. Identifying F2R as a prognostic biomarker underscores its importance in guiding treatment decisions and the development of targeted therapies. Considering F2R expression in treatment planning reinforces its role in personalized treatment strategies. Our analysis of the impact of F2R on clinicopathological parameters in BC patients revealed that elevated F2R expression levels were particularly notable among those with ER + and/or PR + BC. This finding was corroborated by experiments on MCF-7 cells, where F2R levels were significantly higher compared to MCF-12A cells, especially in cells positive for ER and PR.

F2R demonstrates multifaceted mechanisms of action within tumors. For instance, in liver cancer, F2R influences the efficacy of PD-1 immunotherapy through the JAK2/STAT1 signaling pathway [21]. Additionally, in lung adenocarcinoma, F2R has been identified as a promoter of tumor angiogenesis via the EGFR pathway [23].

ECM receptor and PI3K-Akt pathways are pivotal in tumor initiation, progression, and treatment [24,25,26,27]. Enrichment analysis identified correlations between F2R and these pathways. These findings suggest that F2R is involved in BC cell proliferation and invasion, indicating its potential as a target for BC therapies. Exploring the synergistic effects of F2R-targeted treatments with existing modalities, such as chemotherapy and immunotherapy, holds promise for improving clinical outcomes. Such investigations may lead to combination therapies with enhanced efficacy while minimizing adverse effects.

The TME plays a critical role in the survival of tumor cells, significantly influencing their proliferation, metastasis, immune evasion, and treatment resistance [28,29]. Targeting the TME has thus become a central focus in cancer research and clinical practice [30]. Investigating F2R within the TME, particularly its interactions with immune cells, presents opportunities for immunomodulation. Targeting F2R could potentially reshape tumor-immune dynamics, thereby enhancing the efficacy of immunotherapy in BC. Translating these findings into clinical applications is crucial, as it provides evidence on the efficacy and safety of F2R-targeted therapies. This could lead to the development of novel therapeutic approaches for patients with BC.

Tumor immunotherapy has demonstrated considerable efficacy in various solid tumors and is regarded as a groundbreaking advancement. However, the clinical response rates remain relatively low [31]. Immune checkpoints are currently critical for determining a patient’s eligibility for immunotherapy, with TMB being closely associated with immune checkpoint inhibitors (ICIs). High TMB (TMB-H) has been suggested as a predictor of ICI response [32,33]. However, TMB-H has not consistently predicted responses to immune checkpoint blockade across various cancer types, including BC, prostate cancer, and glioma [34]. In the current study, a significant association was observed between increased F2R expression and reduced TMB in patients with BC. Additionally, F2R expression showed a strong positive correlation with immune checkpoint molecules such as CD200 and NRP1. Previous studies have indicated that elevated expression of CD200 and NRP1 can enhance the efficacy of immunotherapy [35,36]. These findings suggest that while TMB may provide some insight, it may be an incomplete biomarker for evaluating the effectiveness of ICIs.

The impact of immune cell infiltration on the prognosis and treatment response of BC is increasingly recognized [37,38,39,40]. The CIBERSORT analysis indicated that high F2R expression in BC correlates with increased levels of memory CD4^+^ T cells, mast cells, dendritic cells, and plasma cells. Conversely, low F2R expression is associated with natural killer (NK) cells, M0 macrophages, and memory B cells. The activation of CD4^+^ T cells, known for their role in modulating cytolytic activities and enhancing the responses of B cells and CD8^+^ T cells, suggests a complex interaction in the progression of BC [41]. The linkage between F2R and immune pathways underscores its significant role as an immune regulator. High F2R expression activates tumor-related immune cells, leading to potentially improved outcomes for patients with BC. Thus, F2R emerges as a critical prognostic marker and a regulator of immune responses in BC.

Drug sensitivity analysis and molecular docking are essential methodologies in drug discovery and development, facilitating the identification of more efficacious therapeutic agents and enhancing treatment precision. The current study revealed a heightened sensitivity of dasatinib to F2R, suggesting its promising application in BC therapy. Additionally, *in vitro* experiments underscored the significant biological impacts of dasatinib on the BC cell line MCF-7, further emphasizing F2R as a potential therapeutic target. These findings emphasize the potential of dasatinib and F2R-targeted approaches in the treatment of BC.

In conclusion, this study advances the understanding of F2R’s role in BC by combining bioinformatics analysis with *in vitro* cell experiments. This integrated approach allows for a more precise exploration of BC pathogenesis and potential therapeutic targets. The findings provide a strong molecular basis and open new avenues for further experimental investigations. However, certain limitations must be acknowledged. The reliance on public databases and published literature may introduce biases, and the quality of data could affect the results. Therefore, rigorous statistical methods are essential for interpreting the findings accurately. Nevertheless, the consistency observed across databases and experiments enhances the reliability of the conclusions. Future research should focus on investigating the cellular biological functions and dasatinib sensitivity of BC cell lines following F2R knockdown or overexpression.

## 5 Conclusion

By utilizing bioinformatics analysis, we investigated the relationship between increased F2R expression and the progression of BC, evaluating its potential as a prognostic marker and its influence on BC-related pathways. The findings underscore key aspects of BC prevention and prognosis by revealing a complex lncRNA-miRNA-mRNA ceRNA network centered around F2R. This network analysis offers preliminary insights into the role of F2R in the early stages and progression of BC. GSEA directly linked F2R-related signaling pathways with tumor progression, further emphasizing its significance. Experimental validations reinforced the potential of F2R as a predictive biomarker and its viability as a target for immunotherapy in BC. Additionally, further research elucidated the biological mechanisms that contribute to elevated F2R expression during BC progression. These findings highlight the importance of F2R in BC pathogenesis and suggest its potential utility in guiding future therapeutic strategies.
